# Mechanical Performance of Polymeric ARGF-Based Fly Ash-Concrete Composites: A Study for Eco-Friendly Circular Economy Application

**DOI:** 10.3390/polym14091774

**Published:** 2022-04-27

**Authors:** Hassan Tariq, Rao Muhammad Arsalan Siddique, Syyed Adnan Raheel Shah, Marc Azab, Attiq-Ur-Rehman, Rizwan Qadeer, Muhammad Kaleem Ullah, Fahad Iqbal

**Affiliations:** 1 Department of Civil Engineering, Pakistan Institute of Engineering and Technology, Multan 66000, Pakistan; ht.awan786@gmail.com (H.T.); raoarslansiddiq225@gmail.com (R.M.A.S.); attiqurrehman975@gmail.com (A.-U.-R.); rizwan44civil@gmail.com (R.Q.); 2 College of Engineering and Technology, American University of the Middle East, Kuwait; marc.azab@aum.edu.kw; 3 Department of Civil Engineering, University of Lahore, Lahore 54000, Pakistan; muhammad.kaleem1@ce.uol.edu.pk; 4 Department of Mechanical and Structural Engineering and Materials Science, University of Stavanger, NO-4036 Stavanger, Norway; fahadmeyo@gmail.com

**Keywords:** polymer, alkali resistance glass fiber (ARGF), fly ash (FA), eco-friendly, mechanical properties, concrete, industrial fibers

## Abstract

At present, low tensile mechanical properties and a high carbon footprint are considered the chief drawbacks of plain cement concrete (PCC). At the same time, the combination of supplementary cementitious material (SCM) and reinforcement of fiber filaments is an innovative and eco-friendly approach to overcome the tensile and environmental drawbacks of plain cement concrete (PCC). The combined and individual effect of fly ash (FA) and Alkali resistance glass fiber (ARGF) with several contents on the mechanical characteristics of M20 grade plain cement concrete was investigated in this study. A total of 20 concrete mix proportions were prepared with numerous contents of FA (i.e., 0, 10, 20, 30 and 40%) and ARGF (i.e., 0, 0.5, 1 and 1.5%). The curing of these concrete specimens was carried out for 7 and 28 days. For the analysis of concrete mechanical characteristics, the following flexural, split tensile, and compressive strength tests were applied to these casted specimens. The outcomes reveal that the mechanical properties increase with the addition of fibers and decrease at 30 and 40% replacement of cement with fly ash. Replacement of cement at higher percentages (i.e., 30 and 40) negatively affects the mechanical properties of concrete. On the other hand, the addition of fibers positively enhanced the flexural and tensile strength of concrete mixes with and without FA in contrast to compressive strength. In the end, it was concluded that the combined addition of these two materials enhances the strength and toughness of plain cement concrete, supportive of the application of an eco-friendly circular economy. The relationship among the mechanical properties of fiber-reinforced concrete was successfully generated at each percentage of fly ash. The R-square for general relationships varied from (0.48–0.90) to (0.68–0.96) for each percentage of FA fiber reinforced concrete. Additionally, the accumulation of fibers effectively boosts the mechanical properties of all concrete mixes.

## 1. Introduction

In the construction industry, cement has been recognized as the utmost energy-intensive material after steel and aluminum [1]. For the manufacture of Ordinary Portland cement (OPC) a huge amount of energy is required; every ton of cement in a typical cement plant consumes around 110–120 kWh energy [2]. Meanwhile, cement poses a significant threat to the environment with the release of about 7% carbon dioxide during its production process [3]. The production of cement can affect the atmosphere in two ways: the first is known as calcination, which involves the direct release of carbon dioxide and the second is indirect release due to the burning of fossil fuels that are required for the heating of kiln, etc. [4,5]. Moreover, to produce one ton of cement, approximately 2.8 tons of raw materials (i.e., limestone, shale, etc.) are required, which is a serious resource depleting procedure (RDP) [6]. Additionally, each year the construction industry consumes at least one trillion liters of water for concrete preparation [7]. Despite all these reasons, still, cement concrete is broadly utilized in the construction industry on a daily basis [8]. Hence, for sustainable economic progress, highly resourceful applications of renewable and non-renewable resources are crucial [9,10]. The foremost concern of this era is to replace Portland cement with a unique material to lessen the resource depletion procedure and save our world from concrete environmental anxieties [11,12].

One of the most reliable techniques to lessen the environmental influence of cement is to replace it with some valuable waste supplementary cementitious material (SCM) such as fly ash (FA). The utilization of SCM not only lessens the energy consumption and resource depleting procedure but is helpful for the eco-friendly utilization of hazardous solid waste [13]. The intention of a circular economy can be accomplished via the utilization of SCM in concrete production [14,15]. To successfully meet the electricity needs in numerous countries of the world such as Pakistan, coal power plants have been installed. FA is easily available in thermal power plants because it is a by-product of pulverized coal combustion. This power plant FA can be suitably employed as a pozzolanic material as it contains silica, alumina, and other dehydrated mineral in large quantities [16]. Numerous scholars have studied the physical, mechanical, and durability characteristics of FA concrete and have concluded that FA concrete successfully enhances the durability properties of concrete such as acid attack resistance (AAR), water absorption (WA), drying shrinkage (DS), etc. [17,18,19,20]. On the other hand, a high percentage of replacements appear to be unfavorable for early-age mechanical properties [21,22], while a very small amount of enrichment can be noted: up to 15% cement replacement [23,24]. Some scholars conclude that the physical and chemical properties of FA (e.g., composition, fineness, carbon content, etc.) critically influence the properties of concrete [25,26,27,28].

The low tensile properties are a drawback of conventional concrete because it limits its structural applications in the construction industry. Generally, the tensile strength of conventional concrete is 12–15-times lower than its compressive strength [29,30,31]. Many scholars excellently answer this problem and advise the utilization of distinct fibers in conventional concrete. For the enhancement of fracture resistance and tensile characteristics of concrete, scholars have suggested numerous fibers such as glass, steel, carbon, polypropylene, polyvinyl fibers, etc. [32,33,34,35,36,37]. Alternatively, the assortment of these fibers mainly depends upon their applications. Instead, a few scholars have estimated that the utilization of industrial fibers is not economical [35]. Some of their drawbacks in conventional concrete include that they lessen the workability of concrete and increase its cost. To lower the workability problem, the addition of good quality plasticizer has been suggested according to the quantity and category of fiber. Consequently, it is essential that these insistent problems are resolved for the promotion of eco-friendly concrete. Through the mutual combination of fiber and SCMs, a novel kind of durable, economical, ductile, and eco-friendly concrete can be obtained. Scholars have found that the blend of these two materials has plentiful benefits such as (i) the efficiency of fibers reinforcement can be enhanced through SCMs because they increase the bond among fiber-filaments and the matrix of the binder [38,39], (ii) the workability problems of fiber-reinforced concrete (FRC) can be minimized through SCMs (e.g., FA and slag) [40,41,42,43], and (iii) the scattering of fiber-filaments can also be improved by SCMs (e.g., slag and silica fume) [44,45]. Hence, the blend of these materials reveals the synergistic performance of the properties of concrete [19]. Several scholars have utilized industrial fibers in their studies, revealing their comportment with waste SCMs [41,46,47,48,49]. Meanwhile, various studies have utilized distinct kinds of fibers (e.g., glass, steel and synthetic, etc.) in conventional concrete to enhance their mechanical properties. Choi and Yuan [31], Ghugal, and Deshmukh [50] investigate the mechanical properties of conventional concrete at the diverse dosages of glass fibers. Similarly, Vijai et al. [51] investigate the mechanical properties of FA-based geopolymer concrete (GPC) at varied percentages of glass fibers (varies from 0.01–0.03% by volume of concrete). They reveal that the addition of glass fibers lessens the mechanical properties of FA-based GPC in contrast to FA-based GPC without glass fibers.

**Figure 1:**
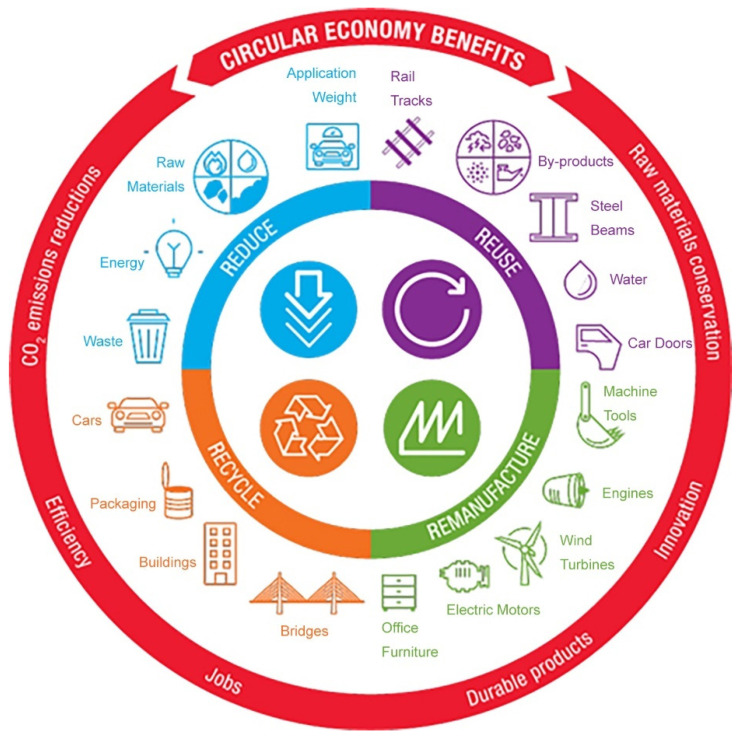
UNCTAD-Perspective Circular Economy Model [52].

The utilization of waste materials follows the circular economy model pattern which covers the concept that waste from one source is a product of another source. The complete cycle of the circular economy model has been described by United Nations Conference on Trade and Development (UNCTAD) as shown in Figure 1. It explains the purpose as “A circular economy benefits both developed and developing countries, keeping materials longer in the economy could reduce by 33% the CO_2_ emissions embedded in products, mitigating emissions at lower costs than other strategies, and helping countries in their Paris and SDGs commitments” [52].

This CE models follow the utilization of waste material for a connection-based relation for eco-friendly infrastructure improvement and economic development.

The prime objective of this study is to analyze the mechanical properties of eco-friendly composite material based on the utilization of FA and ARGF. For this aspect, following laboratory tests, compressive, split tensile, and flexural strength tests were applied. After testing, all results of specimens were compared to conventional concrete graphically, analytically, statistically, and theoretically. This study will follow the eco-friendly development of infrastructures through economical resource utilization.

## 2. Materials and Methods

### 2.1. Basic Materials

The current study involved the following material binders: (i.e., cement, fly ash), sand, coarse aggregates, AR-glass fiber, and water for the development of AR-glass fiber fly ash concrete. The proportions of these materials are designed according to M20 grade concrete. Maple Leaf brand cement with grade 53 of type-1 was utilized as a cementitious material as per ASTM C-150 [53]. The coal-based fly ash obtained from the coal power plant in Pakistan was used as a supplementary cementitious material (SCMs). In Pakistan, the annual production of FA is around 5 million tons which is equal to 10% demand for its OPC clinker [54]. According to ASTM C-618, fly ash is recognized as a class-F of coal ash [16]. The chemical and physical composition of these cementitious materials is listed in Table 1 and Table 2 [55,56,57,58,59].

**Figure 2:**
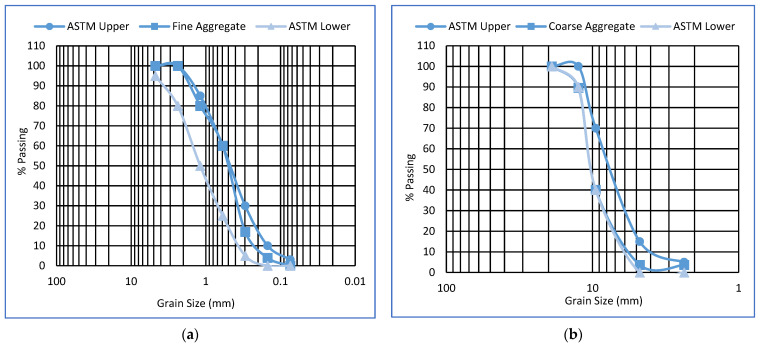
Gradation curve for (**a**) Fine Aggregates (**b**) Coarse Aggregates.

After the description of cementitious materials, the next one is fine and coarse aggregates. Locally available well-graded fine aggregates with a maximum size of 4.75 mm were used in this study. The maximum and minimum size of well-graded coarse aggregates is 12.5 mm and 4.75 mm, respectively, and originated from Sargodha, Pakistan. The physical characteristics of these aggregates are listed in Table 2 as per ASTM standards [60,61,62,63,64,65,66,67]. Tap water is used for the mixing of all these materials. Fine and coarse aggregate gradation curves are shown in Figure 2.

Countless kinds of fibers are available on the market; glass fiber is favored due to its higher ratio of surface area to weight. For this research, alkali resistance glass fiber (AR-GF) was used for the preparation of fibrous concrete, as this type of fiber is not affected by the alkaline condition of the cement [68]. The physical properties of these fibers are listed in Table 3.

### 2.2. Mix Proportion and Specimen Preparation

In this study, the utilization of AR glass fiber has been carried out at four distinct percentages (i.e., 0, 0.5, 1, 1.5) by the volume of concrete. Similarly, fly ash has been added as a replacement for cement at various percentages (i.e., 0, 10, 20, 30, 40) by the volume of cement. M20 grade concrete has been used in this investigation at the w/c ratio of 0.60. The binder (e.g., cement and fly ash), and coarse and fine aggregates were put into a mechanical concrete mixture for 4 min. After the required amount of water was added into a concrete mixer, the machine again ran for 2 min. In the end, the required quantity of fibers was added to this wet-concrete blend and the mixer ran for 5 min only. For the homogeneous mixing of all ingredients, special attention was applied during mixing. Before the concrete mix was put into molds, Abram’s slump-cone test was performed for the measurement of workability of concrete as per ASTM C-143 [69]. The workability ranges from 66 mm to 127 mm for all concrete mixes without fibers, parallel to others that contain fibers ranging from 25 mm to 82 mm. After the interval of 24 h, the specimens were successfully demolded and put into a curing tank at a room temperature of 23 ± 2 °C following ASTM C-192 [70]. The detailed mix proportion for each concrete mix is listed in Table 4 below.

### 2.3. Methods of Specimen Testing

**Figure 3:**
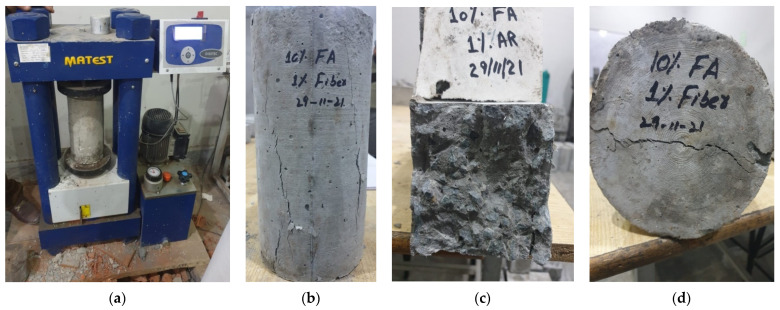
(**a**) MATEST brand testing machine, (**b**) Compression specimen, (**c**) Flexural specimen and (**d**) Split tensile specimen.

In this study, the MATEST brand machine with a maximum loading capacity of 2000 kN shown in Figure 3 was operated for the evaluation of mechanical properties (e.g., compression, flexural and indirect split tensile, etc.) of AR glass fiber-fly ash concrete. The curing of specimens was carried out for 7 and 28 days. For compressive and indirect split tensile strength tests, a cylinder with a size of 150 mm × 300 mm was cast. Meanwhile, beams with a size of 100 mm × 100 mm × 500 mm were cast for the flexural strength test. The curing and testing of specimens were conducted according to ASTM standards. The compressive and indirect split tensile strength test was conducted at the loading rate of 5 kN/s as per ASTM C-39 and C-496 [71,72]. Similarly, the third point load flexural strength test was conducted at a loading rate of 1kN/s as per ASTM C-78 [73]. For a single type of testing, a total of three specimens were prepared for every concrete mix at 7 and 28 days, respectively. Then, the average of the three specimen values counted as the final value of that experiment.

## 3. Result and Discussion

### 3.1. Compressive Strength Performance

**Figure 4:**
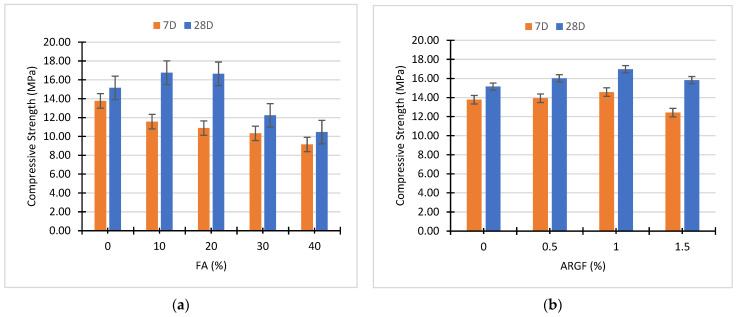
Effect of (**a**) Fly Ash and (**b**) AR glass fibers on the compressive strength of concrete.

After the testing of specimens, the separate effect of fly ash and AR glass fiber on the strength of 7D and 28D concrete is illustrated in Figure 4. The results indicate that the strength of the concrete rises at 10% and 20% replacement of cement with FA. Further replacement declined the strength of concrete at 7 and 28D, respectively, as shown in Figure 4a. However, the highest strength results of 28D curing were obtained at 10% replacement, which equals to 16.76 MPa. At 7D curing, the strength of fly ash concrete decreases in contrast to reference concrete (0% FA). In the case of ARGF addition, the highest strength of 28D curing was obtained at 1% addition of fibers, which equals 17 MPa, as shown in Figure 4b. Similarly, at 7D curing, the fiber-reinforced concrete strength increases compared to the reference concrete (0% ARGF). The lowest strength of 7D curing was obtained at 1.5% ARGF, which is 11% less than the reference concrete mix.

**Figure 5:**
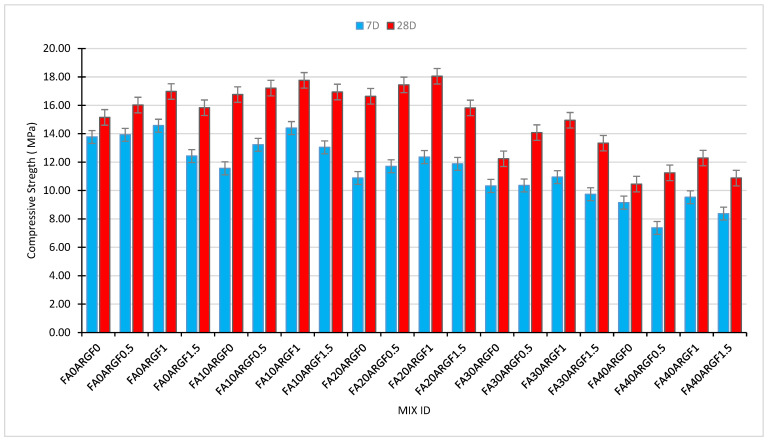
Mutual effect of FA and ARGF on the compressive properties of concrete.

The outcomes presented in Figure 4b demonstrate strong agreement with previous studies conducted by Swami et al. [74], and Hilles et al. [75] determining the behavior of glass fiber reinforced concrete composites. According to Swami et al., a small percentage of fibers significantly increases the compressive strength of concrete. Conversely, the compressive strength did not increase when the percentage addition of fiber was superior to the fibers optimal value, as shown in Figure 4b and Figure 5. In this study, the fiber optimal value can be noted at 1 percent fiber addition, as revealed in Figure 4b. Hilles et al., who used glass fiber to study the mechanical properties of high-strength fiber reinforced concrete, show that the optimal value of glass fiber was achieved at 1.2 percent addition. The mutual effect of FA and ARGF on the compressive properties of concrete at 7D and 28D curing is illustrated below in Figure 5.

Figure 5 briefly illustrates the compressive strength behavior of each mix ID. At 28D, FA10ARGF1 and FA20ARGF1 concrete mix attained maximum compressive strength in contrast to all concrete mixes. In addition, the compressive strength of FA20ARGF1.5 was approximately equal to reference concrete at 28D curing. By the mutual effect of FA and AR glass fiber, the highest improvement was noted at FA20ARGF1. The mixtures that contained only FA did not gain sufficient strength at 7D curing. Furthermore, almost all mixes gained less strength in contrast to reference concrete mix, except FA10ARGF1, which achieves 5% higher strength at 7D curing. Similar behavior was noted in the scenario of FA30ARGF1. At 7 and 28D curing, the compressive strength of FA30ARGF0 and FA40ARGF1 is almost equal after the addition of fibers. The 1% addition of ARGF demonstrated positive results at each percentage replacement of cement with FA.

In terms of discussion, the outcomes proved that the low-level integration of FA (i.e., 10–20%) successfully participates in the pozzolanic reaction of the binder matrix, as verified by Boga et al. [23]. Due to this, compressive strength rises at these percentage substitutions, as shown in Figure 4a and Figure 5. In addition, the compressive strength of fiber-reinforced fly ash concrete was directly subsidized by smaller particles of fly ash. Due to the filling effect, these particles enhance the strength of concrete [26]. In contrast, at a higher-level substitution of fly ash (i.e., 30–40%) the compressive strength of concrete declined, as shown in Figure 4 and Figure 5. Following Akhtar et al. [14], this trend is directly linked with the reduction in the calcium oxide content of the binder, owing to the higher amount of fly ash. Moreover, the performance of fiber-reinforced concrete increases with the addition of fly ash (10–20%) level. All this happens due to the enhancement of the fiber–binder matrix bond, as shown in Figure 5. Meanwhile, high-level incorporation of fly ash (i.e., 30–40%) weakens the fiber–matrix bond and lessens the compressive strength of fiber reinforced concrete. Meanwhile, fly ash concrete gains more strength at later ages due to fly ash bonds with ordinary Portland cement. Additionally, the distribution of stresses to fiber can be affected due to a weaker binder matrix according to Akhtar et al. [14].

### 3.2. Split Tensile Strength Performance

**Figure 6:**
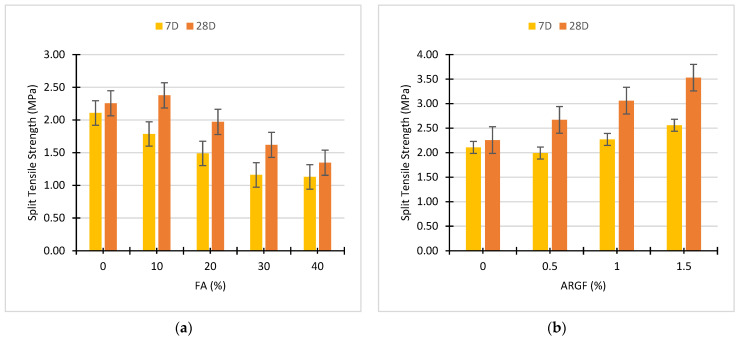
Effect of (**a**) Fly Ash and (**b**) AR glass fibers on the split tensile strength of concrete.

This section discusses and explains the performance of FA and ARGF on the split tensile properties of each concrete mix ID. Firstly, the individual performance of FA and ARGF at 7D and 28D curing is demonstrated in Figure 6. At 28D, the split tensile strength of concrete containing 10% FA increases around 6% compared to the reference concrete mix. Further replacement of cement with FA declines the split tensile strength of the concrete after 28D curing. The impact of fly ash on split tensile strength is negative, as shown in Figure 6a. At 7D, fly ash concrete gains less strength in contrast to the reference mix. Only a minor enforcement in tensile strength is noted at 28D strength of FA10ARGF0 mix ID. Similar findings were reported by Akhtar et al. [14] at 10% incorporation level of fly ash. They stated that further incorporation declined the split strength of concrete. However, owing to slow pozzolanic activity, split tensile strength decreased at a higher-level incorporation of fly ash [23].

**Figure 7:**
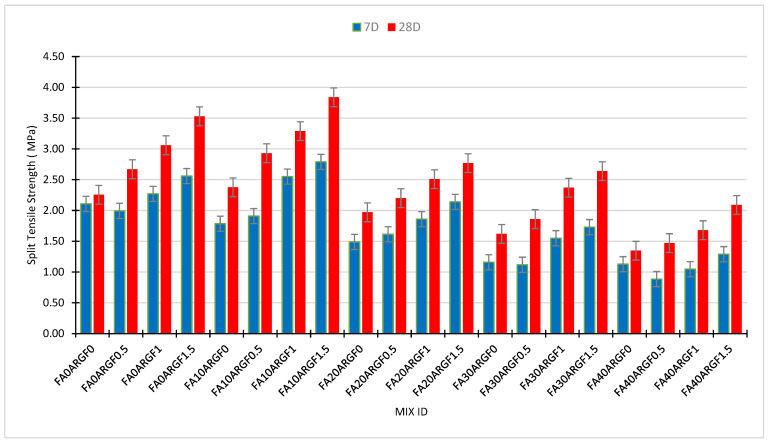
Mutual effect of FA and ARGF on the split tensile properties of concrete.

Figure 6b demonstrates the separate effect of ARGF on the split tensile strength properties of concrete mix at 7 and 28D curing. The 28D highest strength around 3.53 MPa obtained at 1.5% addition of ARGF. This value of 1.5% ARGF concrete is 56% higher than the reference concrete mix. On the other hand, 28D lowest split tensile strength approximately 2.30 Mpa noted at 0% addition of ARGF. Furthermore, 7D strength after the addition of 0.5% ARGF is almost equivalent to the reference concrete mix. All these outcomes show good agreement with previous studies conducted by Hilles et al. [75] and Swami et al. [74], who study the mechanical behavior of glass fiber concrete composites. Hilles et al. reported that the split tensile strength continuously increased as the fiber percentage varies from 0 to 1.2%. It was concluded that reinforcement of 1.2% glass fiber enhances the split tensile strength of concrete by around 63% compared to reference mix strength. Furthermore, Swami et al. reported that the split strength of concrete rises significantly with the addition of glass fiber, even when a large volume was utilized. Current study outcomes shown in Figure 6 and Figure 7 illustrate a good relationship with previous studies. The mutual effect of FA and ARGF on the split tensile properties of each concrete mix at 7D and 28D curing is illustrated in Figure 7.

A brief explanation of each mix ID with or without the mutual effect of FA and ARGF is illustrated in Figure 7. With the addition of ARGF with 0% FA, the 28D split tensile strength varies from 2.67 to 3.53 MPa, and the highest strength was obtained at 1.5% addition of ARGF. The same trend is seen in the case of 1.5% ARGF with 10% FA and the 28D strength varies from 2.93 to 3.84 MPa. The 28D split tensile strength of 20% FA with ARGF ranges from 2.20 to 2.77 MPa and the higher value is obtained at 1.5% ARGF with 20% FA. Compared to reference concrete, mix ID comprising 10% FA and 1.5% ARGF exhibits almost 70% higher strength. This improvement successfully displays the cooperation of the binder matrix with fiber. Over time, the strength of concrete mixes at the age of 28D gains more strength compared to 7D strength results.

With the varying amount of fly ash, the split strength of ARGF concrete changes simultaneously. Akhtar et al. [14] stated that the enhancement of ARGF concrete strength is directly linked with the pozzolanic reaction of the binder matrix. Like for all 10% FA mix IDs, the split strength rises due to the filling effect of fly ash. Additionally, greater than 10% incorporation of fly ash decline the split strength of concrete, but it is greater than the reference mix strength. The minimum strength achieved 40% substitution of fly ash. At 40%, the strength upsurges due to fiber addition because of weaker binder matrix efficiency of fibers, which declines at a higher level of fly ash. The weak binder matrix shows lower bond strength of fiber-matrix compared to a strong binder matrix according to Ali et al. [76]. So, 10% substitution of fly ash is recommended for maximum ductility benefits.

### 3.3. Flexural Strength Performance

**Figure 8:**
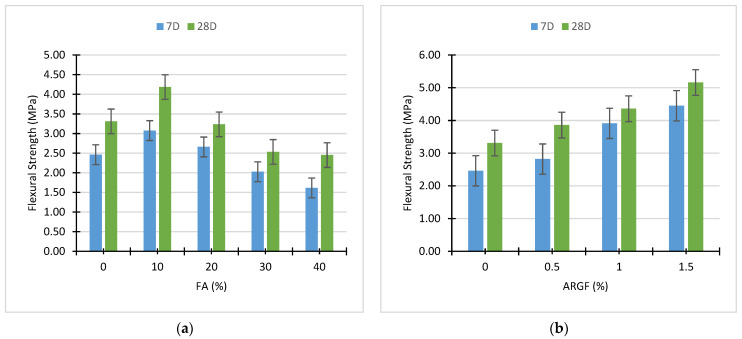
Effect of (**a**) Fly Ash and (**b**) AR glass fibers on the flexural strength of concrete.

This section explains the flexural strength behavior of each concrete mix at the age of 7D and 28D curing. Figure 8 illustrates the individual effect of FA and ARGF on the flexural properties of concrete mixes at both curing ages. The flexural strength of FA concrete is shown in Figure 8a which elaborates their 7 and 28D performance at each percentage of replacement. The results reveal that the 10% FA concrete gains maximum strength as a contrast to all FA concrete mixes at both curing ages. For 30 and 40% FA concrete mixes, the 28D strength results are almost equal. Especially, the strength of reference concrete and 20% FA concrete are equal. This observation was also noted in the outcomes of compression and split tensile strength testing. Previous studies such as Akhtar et al. [14], Ali et al. [76] and Chindaprasirt et al. [26] also reported similar findings for fly ash concrete specimens.

**Figure 9:**
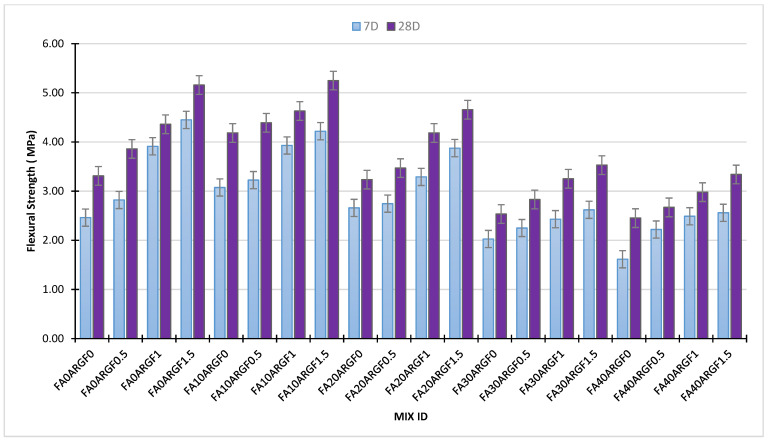
Mutual effect of FA and ARGF on the Flexural properties of concrete.

Moreover, the strength outcomes of ARGF concrete mixes are shown in Figure 8b. At each percentage addition of ARGF, the performance of the concrete mix is successfully improved. The maximum results were obtained at 1.5% addition of fiber that was greater than 5 MPa. The strength of the 1% ARGF concrete mix is almost the same at both curing ages. Current outcomes of ARGF concrete are like previous studies conducted by Hilles et al. [75], and Swami et al. [74]. All these studies reported that, as the percentage of glass fiber rises, the flexural strength of concrete rises simultaneously. Hilles stated that flexural strength enhances around 53% at 1.2% addition of glass fiber. Similarly, in this study, flexural strength rises almost 56% at 1.5% addition of glass fiber. Moreover, the resistance of a material against cracking under tensile load increases after the addition of fiber [74,75,76]. For a better understanding of the mutual effect of FA and ARGF, the outcomes of each concrete mix are illustrated in Figure 9 below.

The combined effect of these two materials, i.e., FA and ARGF, is briefly demonstrated in Figure 9. Additionally, the peak strength was obtained at 1.5% addition of fibers without FA compared to all these concrete mixes. The concrete mixes containing 10, 20 and 30% FA, and 1.5% ARGF exhibit the advancement of flexural strength compared to reference concrete. Additionally, the strength of FA30ARGF1.5 and FA40ARGF1.5 is almost equal to the reference concrete mix strength. From FA0ARGF0 to FA20ARGF1.5, the strength is continuously increased compared to their reference mixes with and without FA at both curing ages. A strong binder matrix with fiber filaments, enhance the flexural strength of concrete. This binder-fiber bond is helpful for the resistance of tensile loads, as described in the above sections. Therefore, these findings are like previous studies [76].

### 3.4. Relationship Analysis among Mechanical Properties

**Figure 10:**
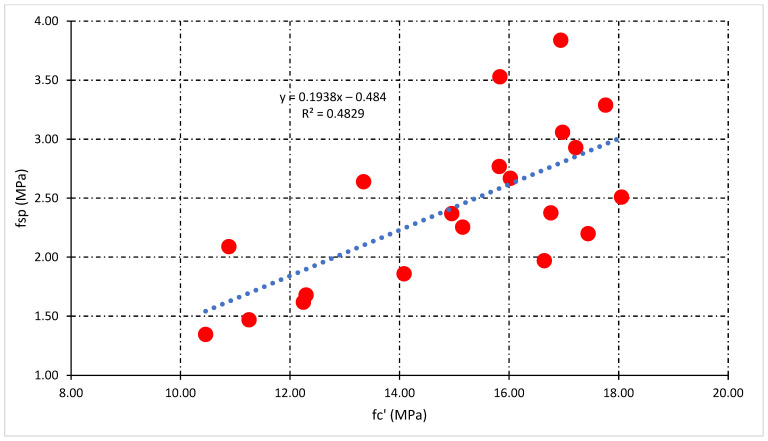
The general relationship between fc’and fsp.

**Figure 11:**
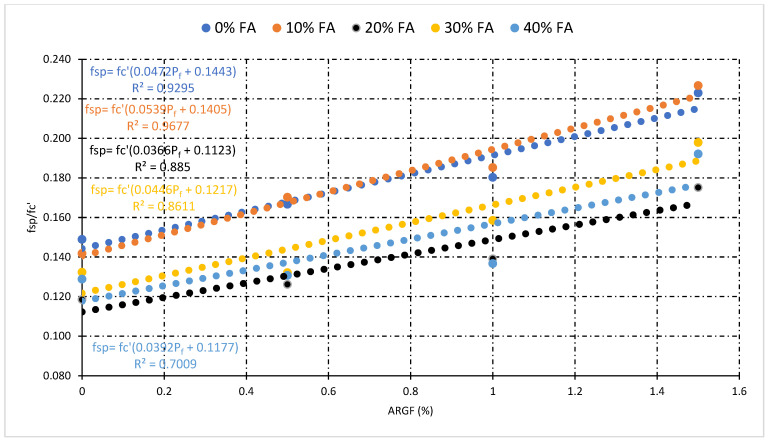
Relationship between fc’, fsp, and percentage of fiber (P_f_).

**Figure 12:**
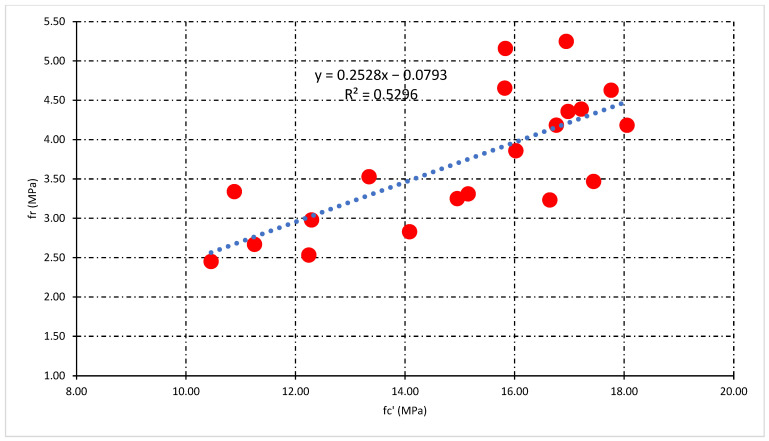
The general relationship between fc’ and fr.

**Figure 13:**
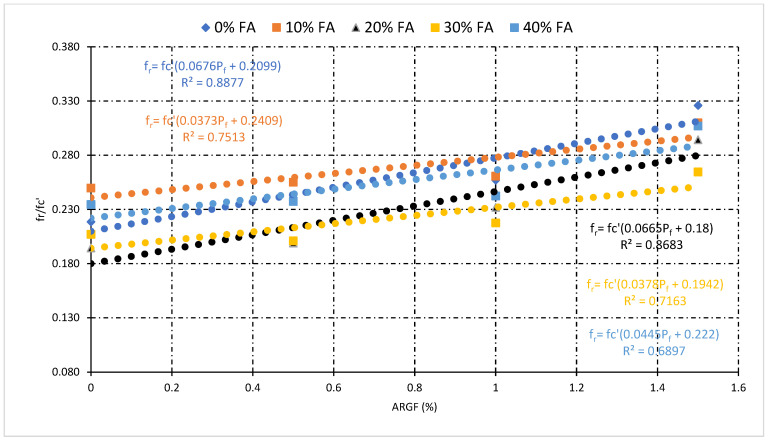
Relationship among fc’, fsp, and percentage of fiber (P_f_).

**Figure 14:**
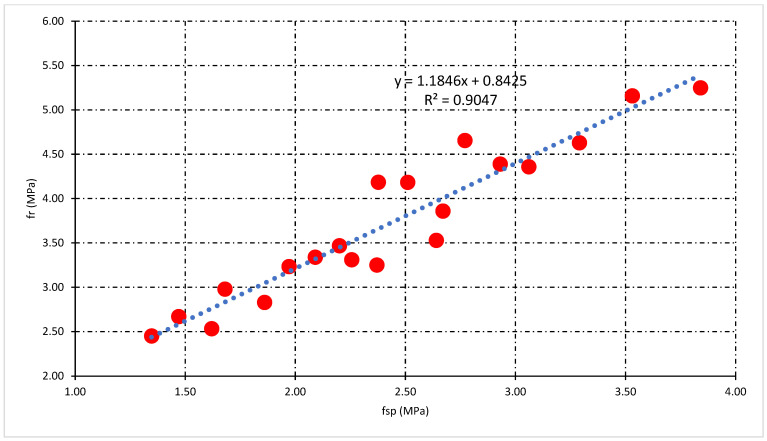
General relationship among fsp and fr for all concrete mixes.

This section illustrates the statistical relationship between the mechanical properties of concrete. The general relationship among compressive strength (fc’), split tensile strength (fsp), and flexural strength (f_r_) for all types of concrete mixes was also investigated. Moreover, the statistical relationship which includes the percentage of fiber (P_f_), fc’, fsp, and f_r_ helpful for the prediction of mechanical properties of fly ash fiber reinforced concrete. Figure 10 and Figure 11 demonstrate the statistical relationship between fc’ and fsp. Figure 10 shows the general relationship for all concrete mixes, but Figure 11 shows the relationship only for fly ash fiber reinforced concrete that included the percentage of fiber (P_f_). Results show that the general relation is comparatively weaker than the relationship generated by the addition of fibers. This confirms the positive effect of fibers on the split tensile properties compared to compressive strength properties. A similar relationship can be developed among fc’and f_r_ demonstrated in Figure 12 and Figure 13. Statistical analysis reveals that the general relationship was found to be very weak among fc’and f_r_ in contrast to the relation that originated by the addition of fibers. In the end, the relationship demonstrated in Figure 14 can develop among fsp and f_r_ for all concrete mixes.

Figure 11 reveals that the highest value of R-square was obtained at 10% FA, while the lowest was obtained at 40% FA fiber concrete mixes. Additionally, the impact of fibers on fsp and fc’ can be tested through this statistical analysis. The above statistical equations will help forecast the mechanical properties of fly ash fiber reinforced concrete. Moreover, in the case of flexural strength analysis, the lowest value of R-square was obtained at 40% FA and the highest obtained at 20% FA fiber reinforced concrete mixes, as shown in Figure 13. However, the overall analysis of mechanical properties of fiber-reinforced concrete was found to be very good. A very strong R-square value of 0.90 was obtained during the general relationship analysis of split vs. flexural strength.

## 4. Conclusions

In this study, the consequence of FA and ARGF on the mechanical properties of concrete were successfully investigated. The result includes that the addition of fibers enhances the mechanical properties of concrete. However, the replacement of cement with FA declined the mechanical properties of concrete at 30, and 40% content. The mutual incorporation of these two materials seriously affects the mechanical properties of all concrete mixtures. The 28D highest compressive strength was obtained at a concrete mix containing 10 and 20% FA and 1% ARGF compared to the reference mix. However, the 28D strength of the reference concrete mix and the concrete mix containing 30% FA and 1% ARGF are almost the same which is considered beneficial for the successful utilization of supplementary cementitious materials in fiber concrete mixes. The concrete mix ID contains 10% FA and 1% ARGF, illustrating maximum 28D split tensile strength in contrast to all concrete mix IDs. In the flexural strength scenario, the optimal strength was obtained at the FA10ARGF1.5 concrete mix. However, the 28D flexural strength of the reference concrete mix and the concrete mix containing 40% FA and 1.5% ARGF were almost the same. The general relationship of mechanical properties for all concrete mixes was not strong compared to the relationship developed by the addition of fibers. The statistical analysis of fiber reinforced concrete verified the positive impact of ARGF on the mechanical properties of concrete. However, the higher level of FA lessens the mechanical properties of fiber-reinforced concrete, i.e., 30 and 40% [14]. While a smaller level of FA can successfully contribute to an increase in strength due to the filling effect [26], a higher level of FA deteriorates the binder matrix that directly reduces the hydration products. As a result, the fiber-matrix bond disturbs the strength of the concrete. All these results express good agreement with other relevant studies.

## Figures and Tables

**Table 1:** Chemical composition of cementitious materials.

Binder Type	LOI	SiO_2_	Al_2_O_3_	Fe_2_O_3_	CaO	MgO	SO_3_	K_2_O	Na_2_O	Miscellaneous
Cement (%)	3.55	20.80	5.02	3.15	62.02	1.86	2.64	0.66	0.42	
Fly Ash (%)	1.79	47.65	23.27	2.16	9.98	1	0.79	____	___	12.26

**Table 2:** Physical properties of concrete ingredients.

Property	Unit	Result		Standard
		OPC	FA	
Density	kg/m^3^	1440	750	ASTṀ C-188
Normal Consistency	%	29		ASTṀ C-187
Specific Surface	m^2^/kg	269	341	ASTṀ C-204
Initial Setting Time	min	127		ASTṀ C-191
Final Setting Time	min	205		ASTṀ C-191
Soundness	Mm	1		BŞ 196-3
Fine Aggregates				
Fineness Modulus	__	2.39		ASTṀ C-136
Bulk Density	kg/m^3^	1440		ASTṀ C-29
Coarse Aggregate				
Bulk Density	kg/m^3^	1530		ASTṀ C-29
Aggregate Impact Value	%	19.35		BŞ 812-3
Aggregate Crushing Value	%	26.50		BŞ 812-3
Los Angeles abrasion	%	29.6		ASTṀ C-131
Water Absorption	%	4.1		ASTṀ C-127

**Table 3:** Physical properties of polymeric alkali-resistant glass fibers.

Fiber Properties	Unit	Results
Tensile Strength	MPa	1700
Modulus of Elasticity	GPa	72
Tensile Strain	%	2
Fiber Diameter	µm	12–20
Bulk Density	kg/m^3^	410
Adhesion to Matrix		Excellent
Alkali Resistance		Good

**Table 4:** Detailed Mix Proportion of This Study In (kg/m^3^).

Mix ID	Cement	Fly Ash	Sand	Aggregate	Water	Fiber
FA0ARGF0	415	0	616	1320	249	0
FA0ARGF0.5	415	0	616	1320	249	3.24
FA0ARGF1	415	0	616	1320	249	6.49
FA0ARGF1.5	415	0	616	1320	249	9.73
FA10ARGF0	373	21.64	616	1320	236.4	0
FA10ARGF0.5	373	21.64	616	1320	236.4	3.24
FA10ARGF1	373	21.64	616	1320	236.4	6.49
FA10ARGF1.5	373	21.64	616	1320	236.4	9.73
FA20ARGF0	331	43.09	616	1320	224	0
FA20ARGF0.5	331	43.09	616	1320	224	3.24
FA20ARGF1	331	43.09	616	1320	224	6.49
FA20ARGF1.5	331	43.09	616	1320	224	9.73
FA30ARGF0	289	65	616	1320	212.4	0
FA30ARGF0.5	289	65	616	1320	212.4	3.24
FA30ARGF1	289	65	616	1320	212.4	6.49
FA30ARGF1.5	289	65	616	1320	212.4	9.73
FA40ARGF0	249	86.36	616	1320	202	0
FA40ARGF0.5	249	86.36	616	1320	202	3.24
FA40ARGF1	249	86.36	616	1320	202	6.49
FA40ARGF1.5	249	86.36	616	1320	202	9.73

FA = Fly Ash, ARGF = Alkali Resistant Glass Fiber.

## Data Availability

Data will be available on suitable demand.
